# Obituary

**Published:** 1909-11-20

**Authors:** 


### Obituary.

The death of Dr. Michael Castaneda, senior consulting physician and late chairman of the Italian Hospital, Queen Square, was announced last week. Dr. Castaneda, who was Chevalier of the Orders of the Crown of Italy and of Charles III. of Spain, spent most of his professional life in London, where he was physician to the Spanish Embassy and to the Italian Consulate. He qualified as a Bachelor of Medicine of the University of London in 1857, and two years later obtained the membership of the Royal College of Physicians.

We have to record the death, on the 13th inst., of Mr. Henry Penfold, surgeon, of Hove. Mr. Penfold took his M.R.C.S. in 1850, and the L.S.A.(Lond.) in the same year. He lived in Brighton itself till recently, and was 81 years of age when he died.

We regret to record the death of Sir William Thomson, C.B., hon. surgeon to His Majesty the King, who died at the age of 64 at his home in Dublin, on November 13. Sir William Thomson graduated M.D. and M.Ch. in 1872, and was a Fellow of the R.C.S. of Ireland, becoming, indeed, its President in 1896, which office he held for two years. He was also a member of the Senate of the Royal University of Ireland (now to be dissolved) and visiting surgeon to the Richmond Surgical Hospital, Dublin. The Mageogh Home and the L. and N.W.R. have also received the benefit of his services. The accession of his present Majesty gave proof of his skill and the services which he had rendered to Queen Victoria as surgeon in ordinary, by appointing him honorary surgeon to the King. In South Africa he gained medal and clasps for his work as chief surgeon to the Irish Hospital. He edited "Power's Surgical Anatomy of the Arteries," and was himself the author of "Ligature of Arteria Innominata", and several articles in the *Trans. Acad. Medicine Ireland*, of which he was general secretary and editor.

